# The reflective component of the Mellow Bumps parenting intervention: Implementation, engagement and mechanisms of change

**DOI:** 10.1371/journal.pone.0215461

**Published:** 2019-04-16

**Authors:** Katie Buston, Rosaleen O’Brien, Daniel Wight, Marion Henderson

**Affiliations:** 1 MRC/CSO Social and Public Health Sciences Unit, University of Glasgow, Glasgow, United Kingdom; 2 Glasgow Caledonian University, Psychology, Social Work and Allied Health Professionals, School of Health and Life Sciences, Glasgow, United Kingdom; Monash University, AUSTRALIA

## Abstract

Understanding why parenting programmes work or do not work, and for whom, is crucial for development of more effective parenting interventions. In this paper we focus on a specific component of Mellow Bumps: reflection on one’s own childhood/past/life. We explore how this component was implemented, how participants engaged with it, the facilitating and constraining factors shaping this, whether and how it appeared to work, or not, and for whom. The paper analyses data from the Process Evaluation of the Trial of Healthy Relationships Initiatives for the Very Early years, which is evaluating two antenatal interventions delivered to vulnerable women, one of which is Mellow Bumps. Data were collected from January 2014 to June 2018 for 28 groups, 108 participants and 24 facilitators in a comprehensive and rigorous Process Evaluation designed to complement the Outcome Evaluation. Data were gathered at various time points using multiple methods, and were synthesised to triangulate findings. The reflective component was implemented with fidelity and participants engaged with it to varying degrees, dependent largely on the coherence of the group. Patchy attendance compromised the coherence of some groups, with the development of rapport, which is key to delivering reflective exercises, more difficult when group composition varied from week to week. Where there was a coherent group, powerful mechanisms of change, leading to stress reduction, included: relief through unburdening, empowerment through support given and received, reduced isolation through sharing anxieties, and control through self-care advice. A minority of highly vulnerable mothers seemed not to benefit from the reflective exercises and were marginalised within their groups. In order to minimise potential harmful effects of such exercises, allocation of participants to groups should strive to maximise group homogeneity. More research is needed to explore how very vulnerable parents can be supported in attending parenting interventions from start to finish.

## Introduction

Group based parenting interventions have been widely identified in policy arenas as important in supporting parents and influencing their parenting behaviours [1, 2]. They have been shown to be effective in modifying aspects of parenting behaviour, and in improving a variety of child and parent outcomes [3–5]. Delivering such parenting interventions during pregnancy offers the opportunity to address parenting behaviours before some of the participants become parents, and at a liminal time in many of their lives. Such timing is also important in light of the evidence of effects on children of stress in mothers during pregnancy [6]. However, there is little evidence on whether it is particularly beneficial to deliver parenting interventions during the antenatal period [7–10].

Furthermore, while there is a large body of work focusing on the efficacy of group based parenting programmes generally, there is less work on effectiveness when implementing on a large scale [11, 12]. Lewis (2011) [13], for example, identifies problems in implementing evidence-based parenting programmes as intended when they are delivered in settings with varying real-world delivery contexts. How participants engage with the interventions as they are delivered has also been less often studied, though Breustadt and Puckering outline barriers to women’s engagement with Mellow Bumps (MB) in their small-scale qualitative study [14]. Precisely how parenting interventions, and particular components of them, work has also received little attention with links between contextual variables, mechanisms and outcomes often oblique [15, 16], Understanding why programmes, as implemented, work and for whom is key in order to develop more effective group-based parenting interventions, that can improve the health and wellbeing of parent and child.

MB is a six week group antenatal programme that aims to reduce maternal stress in pregnancy and encourage attachment with the baby. In this paper we focus on one specific component of MB: reflection on one’s own childhood/past/life. The theoretical rationale for this component is the importance of giving participants the opportunity to talk about issues which may be making them anxious, within the group setting. Doing so in a nurturing and supportive environment is theorised as reducing stress amongst the women, leading to improved maternal and child outcomes. Future papers from the Trial of Healthy Relationships Initiatives for the Very Early Years (THRIVE) study will address the effectiveness of MB as a whole. This paper hones in on MB’s reflective component since it is considered core to the intervention by the programme’s developer(s) and might be a means of modifying the intergenerational transmission of parenting, a key challenge in many parenting interventions.

Other widely used and established group based parenting interventions that incorporate reflective work include Steps Toward Effective and Enjoyable Parenting [17] and Circle of Security [18]. Such work is theorised as changing parenting behaviour in two primary ways: (i) through increasing parental wellbeing via the sharing of stories with other participants in the group, the creation of social bonds and reduction of isolation as well as via empowerment through the process of talking through issues [19, 20] and/or (ii) through increased empathy with their child as guided reflection and discussion enables the parent to understand the child’s emotions and feelings better through remembering her/his own emotions/feelings during childhood [21]. Such reflective work has been seen as having particular utility for vulnerable parents, who are often portrayed as hard-to-reach by those involved in developing and delivering parenting work, but who may particularly benefit from the opportunity to reflect on their childhood and past in relation to their current parenting [20, 22, 23].

Barriers to implementing reflective work have already been identified. Primarily, individual parents may be unable, or may not wish to, reflect on how they were parented, for a variety of reasons. This could be because of her/his intellectual capacity, personality, past experiences, particularly if leading to borderline personality disorder [24], and/or current life, with vulnerabilities in any of these areas often inhibiting willingness or ability to reflect [22, 23, 25]. The characteristics, style and skills of the facilitator may also shape the extent to which individual parents are willing and/or able to engage in reflection within the group. Creating a conducive group atmosphere is necessary, but is skilled work [26, 27]. The nature of the group itself is also relevant, with even the most skilled facilitator finding some group dynamics difficult to manage; this is key as participants’ comfort is dependent on their sense of belonging in the group [28].

By focusing on the reflective component of MB, the paper aims to outline how it was implemented, how participants engaged with it, the factors facilitating and constraining implementation, and whether and how it appeared to work, or not, and for which participants. A large triangulated data set is drawn on to explore these questions, and to identify and discuss potential mechanisms of change amongst the participants. The objective of the paper is to elucidate how and why the reflective component of MB might work in terms of enabling participants to adopt more positive parenting behaviours, including identifying the likely limits to this when considering implementation of the intervention in a real world setting.

### THRIVE

This paper analyses data from the Process Evaluation (PE) of THRIVE, a Randomised Controlled Trial (RCT) of two antenatal interventions, one of which is MB [29]. Recruitment for THRIVE ended in May 2018, and all baseline and PE data collection is complete. Follow-up data collection for the primary study outcomes will continue until May 2019 and these results will be published when available. PE data have been collected using multiple methods over a period of 54 months, for a large number of MB groups, participants and facilitators, in order to elucidate the issues outlined above. While it is becoming more common to conduct PEs to complement Outcome Evaluations (OE) in RCTs of interventions, in line with Medical Research Council Guidance [30], this PE has benefitted from sufficient funding and ample scope to rigorously investigate how the interventions work and for whom. This will enable us to reach important and novel conclusions around the real world implementation of parenting work. This will be of use to policy makers funding and targeting parenting interventions, facilitators and their managers implementing and delivering this work, and to those involved in development of parenting interventions. This paper is the first of a series that will contribute to this broad aim.

THRIVE is a three-arm RCT evaluating two antenatal interventions, Enhanced Triple P for Baby (ETPB) plus Care as Usual (CAU) and MB plus CAU, with reference to CAU [29]. Women were recruited to THRIVE on the basis of additional health and social care needs in pregnancy (e.g. women with a history of substance misuse, domestic violence, mental illness, being looked after in local authority care or criminal justice involvement) using National Health Service Greater Glasgow & Clyde Special Needs in Pregnancy protocol [31]. They were recruited from NHS Greater Glasgow and Clyde, NHS Ayrshire and Arran and NHS South Lanarkshire, in the highly populated Central Belt of Scotland. Stratified block randomisation was used to allocate the women to one of the three arms of the trial. Data analysed for this paper were collected for the trial’s PE, which complements the OE [32]. The focus of this paper is the MB arm.

### Mellow bumps

MB is one of a range of parenting programmes developed by Mellow Parenting (https://www.mellowparenting.org/). It focuses on the antenatal period. MB draws on psychological and practical techniques to reduce anxiety and promote wellbeing in vulnerable pregnant women. A qualitative evaluation found that the strengths of the MB programme include the facilitator’s provision of support, the social aspect of meeting other mothers-to-be, learning about infant development, and receiving practical advice. It identified barriers to engagement as: negative preconceptions about antenatal support, fear of being judged, and feeling pressured by services to participate [14]. A review and meta-analysis concluded that there was some support for the effectiveness of Mellow Parenting for families with multiple indices of developmental adversity [33].

**Fig 1:**
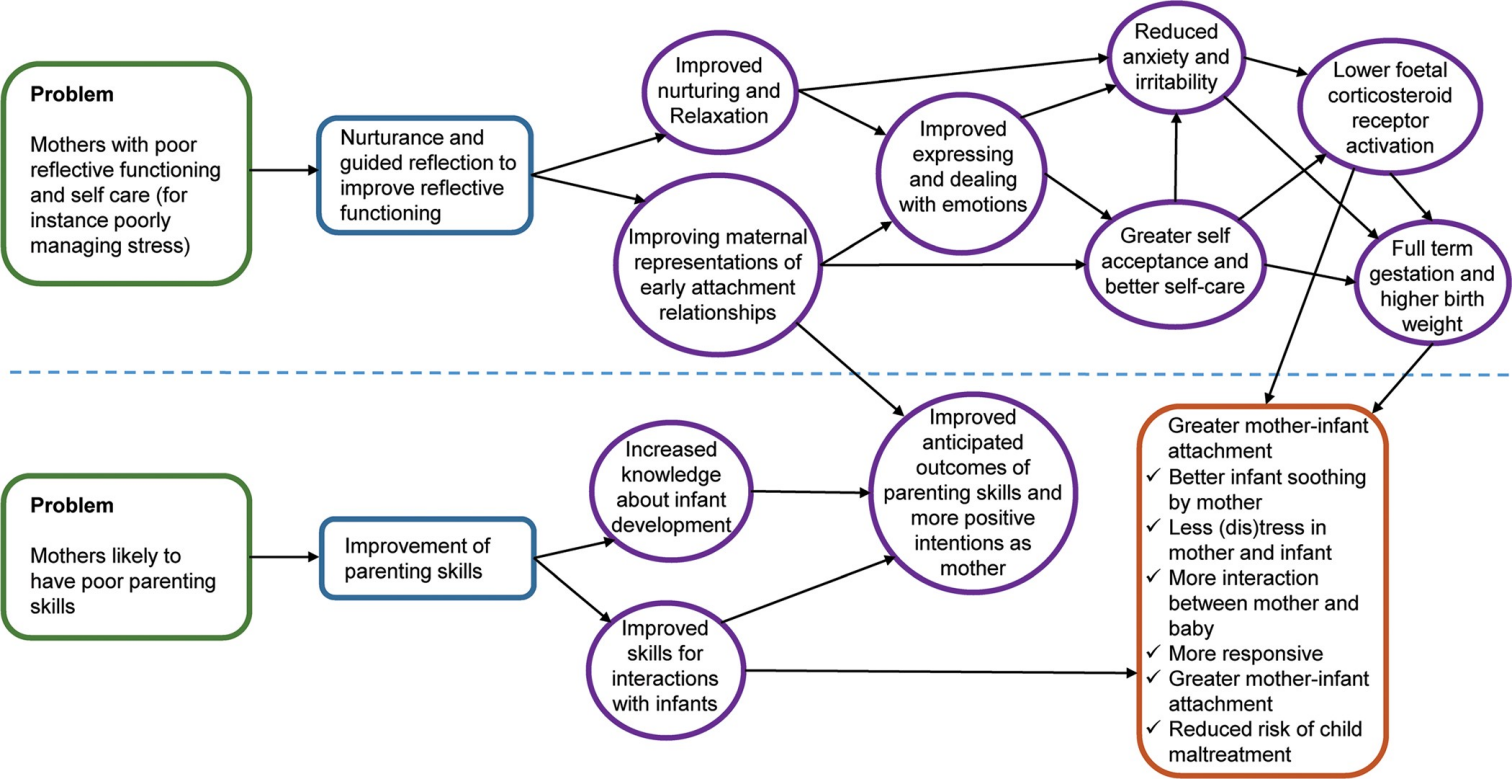
Mellow bumps theory of change.

Fig 1 outlines the theory of change articulated by THRIVE co-investigators, led by MH, in consultation with the developers of MB [32]. It can be seen that ‘guided reflection to improve reflective functioning’ is core to this model.

MB is underpinned by attachment theory and aims to encourage nurturing and attuned relationships between mother and baby. For THRIVE each MB group was delivered by two facilitators, largely midwives, health visitors or family support workers trained in MB delivery. The MB training manual and facilitators’ material is owned by MB and is not publicly available (see <https://www.mellowparenting.org/our-training>). MB groups commenced when participants were between 20–30 weeks pregnant. Groups comprised between three and seven women at the programme start date, with four to ten being the optimal group number. Focused on supporting families with additional needs, MB aims to reduce maternal antenatal stress levels, helping reduce irritability and anxiety, and to increase expectant mothers’ understanding of the capacity of their new-born babies for social interaction. It emphasises the importance of early interaction in enhancing brain development and attachment. Reflection is a less prominent component in MB than in Mellow Parenting interventions for mothers with older children as the intention is to help mothers contain anxiety, and there is recognition that discussing past experiences could actually increase anxiety among pregnant participants. Such work is, however, core to key exercises in two of the six MB sessions. Understanding and empathising with the bump/baby, being attuned with her/his emotions and the importance of nurturing both oneself and the bump/baby are central. Participants are encouraged to identify potential stressors and consider how to manage these, reflect on and identify sources of positive social support, and identify barriers to good parenting. Building a conducive atmosphere and strong relationships within the group are regarded as key by the intervention developers, as are flexible, non-didactic approaches. This is in order to allow a focus on particular issues that are important to the women. The groups are conceived as being nurturing safe closed spaces, in therapeutic terms, providing an environment for manageable contained activities [34]. Taxi transfer to and from each group, held in accessible settings such as hospitals or bookable rooms within supermarkets (part of their corporate social responsibility scheme), was arranged for the women participating in THRIVE.

See Table 1 for an outline of the six or seven two hour sessions (plus pre-meet) included in the MB programme, as set out in the facilitator handbook [34].

**Table 1:** Content of Mellow bumps programme by session.

Session	Content
**Pre**	Meet and Greet: facilitator(s) visit participant at home
**1**	Put participants at ease; establish safe environment; dispel false myths about motherhood; normalise reactions to babies if these are not completely positive; introduce capacities of babies; reduce maternal stress
**2**	Reinforce the acceptance of participants in groups; promote healthy eating, exercise and behaviour; further explore capacities of babies and how parents can respond; reduce maternal stress
**3**	*Address past interferences or support for being a parent*; understand brain development in utero and in the neonate; reduce maternal stress
**4**	*Allow participants the opportunity to explore issues from their past which may impact on their future*; explore and discuss babies’ communication; promote sharing between participants and supporters; reduce maternal stress
**5**	Outing or activity
**6**	Plan future support for mother and baby; make an enduring link in minds of participants; evaluate group; end group positively; reduce maternal stress
**7**	Optional: session involving fathers/partners

Text in italics indicates the session content most relevant to this paper

An outline of activities for each session is also provided in the manual [34], with resources provided for these. *My Island* is a core activity in Session 3, which focuses on what might help or interfere in getting on with the baby. This involves making an island and deciding who will, and will not, be on it so that helpful and unhelpful factors, situations and people in mothers’ lives can be considered. Probably the most pertinent activity for this paper is suggested for inclusion in Session 4. *Ghosts from the Past* involves writing down, considering, and reflecting on problems or events from the past that will make it difficult to be a good mother, and sharing these if the participant is comfortable in doing so.

As with all interventions, practical factors may prevent the intervention being implemented in full or delivered as intended.

## Materials and methods

Table 2 shows the methods used to collect the data analysed for this paper, and the nature of these data. Further detail, including on data sources, can be found here [32]. Interviews and observations were conducted by RO, an experienced qualitative researcher. Two hundred women were randomised to the MB arm of the trial with 176 eligible for groups; 108 attended at least one session. At the time of writing, data were not yet available specific to arm of the trial, due to blinding, but the average age of THRIVE participants was 26 years, and 55% were first time mothers. Preliminary analysis showed high rates (compared to general population) of reported childhood sexual abuse (20%), having spent time as a Looked After Child (31%), social work involvement with pregnancy (36%), and experience of illicit drug use (50%), interpersonal violence (40%) and homelessness (39%) [35].

**Table 2:** PE data: Method, timing, number and nature of data.

Method^a^	When	Number	Data analysed for this paper
Time 1 interviews with mothers allocated to MB arm of trial [T1part]: in-depth semi-structured interviews	After delivery of antenatal component of MB, before birth of baby	9	Background of mothers; experience of attending MB sessions (relationship with facilitators/other group members; group dynamics; benefits/harms of MB)
Time 2 interviews with mothers allocated to MB arm of trial [T2part]: in-depth semi-structured interviews	3–12 months after birth of baby	10 (3 with T1 women)	Background of mothers; reflections on participating in MB, legacy of MB
Time 1 interviews with MB facilitators [T1faci]: in-depth semi-structured interviews	Pre-training	5	Professional background, feelings about delivering MB, how they envisage group delivery
Time 2 interviews with MB facilitators [T2facil]: in-depth semi-structured interviews	Following delivery of MB groups	2 (both with T1 facilitators)	Experiences of delivering groups, understanding of session aims, fidelity, challenges, barriers to participation, who sessions/interventions worked best for
Observation of facilitator training courses for MB [obstrain]: detailed fieldnotes	Training day prior to MB groups being delivered and refresher training day 1.5 years after original training	1 initial (n = 10 facilitators); 1 refresher (n = 4 /original 10 facilitators)	Presentation of intervention theory of change; facilitator views on delivery
Participant observation of MB delivery [obs]: detailed fieldnotes	Throughout delivery phase of THRIVE	Entire delivery of all sessions to 3 groups (G1 n = 5, G2 n = 4, G3 n = 6 participants attending at least one session)	Sessions delivered, fidelity, attendance, facilitator style and relationship with other facilitator and group, participant interaction, level and nature of participation, reaction to materials delivered, atmosphere in group
Session evaluation forms (one per session) [evpart]: brief questionnaire self-completed by MB participants	Throughout delivery phase of THRIVE	115 forms across 27 groups	Comments relating to Session 3 and Session 4 in which *My Island and Ghosts from the Past* are scheduled to be delivered
Session evaluation forms (one per group) [evfacil]: brief questionnaire self-completed by MB facilitators	Throughout delivery phase of THRIVE	43 forms from 9 facilitators across 26 groups	Comments relating to Session 3 and Session 4 in which *My Island* and *Ghosts from the Past* are scheduled to be delivered; any other comments relating to reflective work

^a^Wording in square brackets denotes the abbreviation used to refer to this data source in the main body of the paper.

Twenty four facilitators were trained in MB delivery; nine of them delivered at least one group. They ranged in age between 29 and 55 years, at time of training, with an average age of 42. Only one had had previous experience of delivering MB, though all had experience of facilitating group work. Three were midwives, two were health improvement practitioners; other jobs held included trainee health psychologist and nursery nurse. Twenty eight MB groups were delivered.

A cohesive and robust picture of participants’ and facilitators’ experiences of the reflective component of MB has been built up through triangulation of the data. For example, we have linked group observations to in-depth interview data from some of the participants and the facilitators from those groups in order to examine experiences from the perspective of both. Further triangulation between these data sources and facilitator and participant evaluation forms built the analysis further, with, for some elements of the analysis, other sources such as observational facilitator training data also contributing. These sources have also been linked as part of the analysis in order that multiple perspectives can be incorporated. As such, the rigour of the analysis is high.

First level coding was conducted by RO, across the data-set. KB then focused on analysing the data pertinent to the aims of this paper, i.e. data focused around parental reflection on childhood/past. The initial coding frame for these analyses was formulated by drawing heavily on a literature review conducted by the authors (KB, RO, DW, unpublished), which identified the mechanisms central to reflective work in parenting interventions. The themes that emerged from this review were used to further code the reflective THRIVE data, supplemented by other iteratively derived themes emerging from the data as they were categorised, sorted and analysed. Codes included: nature of reflection, organisation of sessions, feelings about reflecting on past (participant), comments on facilitation (participant), group context, and views on utility/appropriateness of reflective work (facilitator). RO’s first level descriptive coding was validated by KB, and KB’s finer, more explanatory level coding was validated by RO. Cross referencing across data sources also allowed analysis by MB groups.

Interview quotes are described with pseudonyms for the participant or facilitator, and the data source (abbreviations in Table 2 e.g. T2part for Time 2 participant interviews). If the participant being cited was part of one of the groups observed, this is also noted in the parentheses following the quote. Where particular sub analyses were undertaken focusing on a single data source this is highlighted in the results. Generally, however, reported results were generated from a mix of methods, for example group observation data were analysed and were compared with interview data with participants in each group and with the interviews with the facilitators delivering the groups. This enabled us to develop multi-faceted descriptions and explanations.

Data are reported in accordance with the COnsolidated criteria for REporting Qualitative research (COREQ) checklist [36]. Ethical approval was granted to conduct this research by the NHS’s West of Scotland Research Ethics Service, reference 13/WS/0163. Letters of access were provided by NHS Greater Glasgow and Clyde, Ayrshire and Arran, and South Lanarkshire. Informed written consent was obtained from all participants prior to data collection.

## Results

The extent of implementation of the reflective work and participants’ engagement with it is discussed first, before going on to explore facilitating and constraining factors shaping this, identifying key contextual variables. How, and for whom, the reflective work might be effective is then explored in the final section which focuses on mechanisms of change, and how these might bring about the theorised outcomes.

### Implementation of reflective work and levels of engagement

A small number of facilitators expressed reservations around opening a “Pandora’s Box” by delivering the reflective work:

“maybe just touch them with it and see how they react and maybe even just get them to do a bit of it, but I wouldn’t have them really upset, that they couldn’t think 'oh, I can’t do this' and they're … you know, it's really hard to pull them back again” [Andrea, T1facil].

In practice, however, it was largely delivered as set out in the MB manual. Detailed session evaluation data were returned for 26 of the 28 groups delivered (for the remaining two groups neither of the facilitators returned the session evaluation form, despite follow-up by the THRIVE administrator). In all 26 groups *My Island* was delivered. In 25 of those groups *Ghosts from the Past* was delivered, though in one of these groups Session 4 was merged with Session 5 due to the group not being able to go ahead one week, so the exercise was not delivered in full [grp102]. For the group in which it was not delivered at all, facilitators stated:

“[it] was difficult for participants so we decided not to do this” [Christine, grp201evfacil],

“[participants] declined to discuss this [*Ghosts from the Past*]” [Kitty, grp201evfacil]

For *My Island* there were 21 positive and nine negative comments from the facilitators relating specifically to its delivery and how it was received and engaged with by the women [evfacil]. For *Ghosts from the Past* there were 27 positive comments and 16 negative comments. The positive comments related to: the work being effective in facilitating personal disclosures (n = 32) (for example: “Participants really got deeply engaged in ghosts from the past. I was initially apprehensive about how this sessions would go for the participants. It may be too deep and they may not feel ready to open up about past situations. However, I was surprised at how much the girls got involved” [Sally, grp117evfacil]; the women supporting each other during discussions (n = 11); laughter/fun/enjoyment produced by work (n = 3); and the women relating to the work and finding it interesting (n = 2). The negative comments were around: it being problematic (dominating, bored, disinterested or unengaged participant(s)) (n = 8) (for example: “[name of participant] seemed disinterested in other’s experiences. Can be a struggle to engage this particular participant, not interested/arrogant in her responses” [Janice, grp113evfacil]; the work upsetting or increasing stress in participant(s) (n = 8); the women being reluctant to reflect (n = 6); the group being too small (n = 2); and disclosures made that the facilitator found difficult to respond to (n = 1). Most of the negative comments related to individual women in particular groups.

From the perspective of the participants, for *My Island* there were 55 positive comments and 15 negative; for *Ghosts from the Past* there were 28 positive comments and 9 negative [evpart]. The positive comments were around: enjoying talking, sharing and listening (n = 62) (for example “[I liked] learning about everyone’s past and how it makes us” [evpart]); and the content being helpful/ impactful (n = 21) (for example “I felt like I could talk a lot about my anxiety and get some feedback” [evpart]). The negative comments were around: the work not being long/detailed enough (n = 12 (for example “I felt [Ghosts from the Past] could have been more detailed for a longer time” [evpart]); being too negative (n = 4); other participants spoiling atmosphere (n = 2); group too small (n = 2); not relevant (n = 1); uncomfortable room (n = 1); low attendance (n = 1); and the work provoking anxiety (n = 1).

Having the formalised reflective work in weeks 3 and 4 of the programme was recognised by facilitators and participants as important, as the women were more at ease in the group by then:

“It [reflective work] can’t come in at the beginning. It needs to come in at the end of it or halfway through it.[when the women are more relaxed and chatty]” [Andrea, T1facil].

The reflections prompted by *My Island* and *Ghosts from the Past* sometimes included participants’ childhood/past and some reflections were deeper than others. The level of discussion was sometimes described as intimate, and at other times much less so with more superficial issues being shared. An example of deeper reflection was in the first group observed [G1obs,grp105, Janice/Sally facil] where one of the women went into detail about the relationship she had with her mother, and childhood memories of her mother’s alcoholism. She described cutting off contact with her mother and the guilt this had induced, and how it was interfering with her thoughts of her own parenting. The facilitators used this to prompt group discussion around the importance of focusing on oneself, of not feeling guilty, and of getting rid of destructive elements in one’s lives. Deep reflection in the third group observed [G3obs,grp122, Kathy/Sally facil] included Mary opening up about her very difficult childhood, troubled relationship with her parents, and being homeless and heroin addicted at age 12. In this group, in contrast to G1, the facilitators responded to this and other disclosures by providing solutions to the problems identified, rather than opening up a general discussion in the group.

Overall the evaluation forms, backed up by the interview and observational data collected, suggest that the reflective work was implemented in a felicitous way, facilitators found it straightforward and liked it, and most participants present at these sessions appeared to find it acceptable and engaged with it to at least some extent. They enjoyed it and found it useful.

### Facilitating and constraining factors shaping implementation of, and engagement with, reflective work

#### Implementation

Attendance patterns for the reflective work appeared to be the factor key to understanding how the facilitators implemented the reflective work. Seventy of the 108 women who attended at least one session of MB attended Session 3, and 66 attended Session 4. Only 44 women attended both Sessions 3 and 4, and only 29 women attended all four of the first four sessions. Sessions 3 and 4 were attended by fewer women than the other four core sessions (94 women attended Session 1, 82 attended Session 2, 72 attended Session 5 and 71 attended Session 6), Interview data from the participants (T2int) suggests that non-attendance of particular sessions was due to week to week happenings, such as: a sick child, feeling unwell, or a sofa delivery, rather than any anticipated negative feelings around the content of the session. Indeed, there was little, if any, detailing of what future sessions would involve in week 1, or discussion of what would follow in the subsequent week at the end of each previous sessions [obs]. Attendance rates did not particularly vary by facilitator(s) delivering MB. Only four groups ran with four or more women for Session 3 and only three groups ran with four or more women for Session 4. However, the facilitators appeared to be content to work with the low numbers in many of their groups, and implemented the exercises even if attendance was low. Indeed, low numbers were sometimes seen as advantageous:

“[Session4 was a] relaxed, chatty session. Two mums engaged well and opened up around some issues. Easy group to work with as only two mums who seemed relaxed and engaging well” [Janice, grp105evfacil].

Only two of the facilitators explicitly identified that it was problematic to implement the work when attendance was very low.

Lack of group coherence was, however, identified as a key problem when delivering the reflective work, and this was related to the patchy nature of attendance. Working with slightly different groups of women each week, as was typical across the groups, created frustration for some facilitators who felt that they had to create rapport anew. This rapport was particularly important for facilitating the reflective component. Facilitators were faced with situations such as the women bonding well in Session 2 but then key members not coming to Session 3, causing the facilitators to feel that they were going in ‘cold’ working with the group. This was largely the backdrop to facilitator concern around individual ‘problematic’ group members and general reluctance of one or more participants to engage with the reflective work. However, the facilitators worked with this and implemented the reflective exercises anyway, presumably with an awareness of the lack of group coherence. In groups where there was consistent attendance, the facilitators reported that the reflective work was more straightforward to implement [evfacil, T2facil]. Janice, for example, contrasted the third group with the first and second she delivered. In the first two groups the women dipped in and out of attending on a weekly basis, such that a different combination of women attended each week. For this third group {grp106] the same three women attended each week, and the fourth only missed one session. Janice commented that it was much easier for the women to bond with each other and to feel comfortable in this group, and that this context facilitated the successful delivery of the reflective work:

“This group was by far the easiest to work with” [Janice, 106evfacil].

There were also more minor problems and frustrations reported in implementing *Ghosts from the Past*, in particular, in the initial group delivered by some facilitators. This included not judging the time needed to complete the exercise well, and being hesitant in encouraging the women to share their stories. Experience in having delivered MB before (either before THRIVE in the case of one facilitator, or in their first delivery of MB for all the other facilitators) did not, however, appear to shape implementation in any important way.

#### Engagement

There were a number of inter-related factors which shaped participant engagement with the reflective work: coherence of group, bonding, sense of shared commonality, women’s past, facilitator style, unamenable fellow participants, group size and own preference around participation level.

A sense of having bonded with the other women in the group appeared to be a pre-requisite for the women feeling able to comfortably reflect on their childhood/past within the group. Consistent attendance facilitated this, as did ‘feeling a connection’ which, to a large extent, was built on feeling they shared things in common. The women most engaged with the reflective work were those who felt ‘part of the group’. For example, in the first group observed [G1obs, grp105, Janice/Sally facil] the group did not gel particularly well. Sally felt that the reflective work lacked depth because of difficulties the women had building bonds due to fragmented attendance. On the other hand, in the third group observed [G3obs, grp122, Kathy/Sally facil] attendance was high and much more consistent. The facilitators felt that they were delivering MB, by Sessions 3 and 4, to a coherent group in which participants had bonded almost immediately. One participant had shared intimate details about her life during Session 1, with the other women warming to her as a result, showing support and setting the tone of the group which persisted across the weeks, providing the backdrop to Sessions 3 and 4. Indeed, in both Sessions 1 and 2 much of the time was spent with the participants interacting and bonding, in a way not observed in G1 [grp105], where interaction was much more stilted. *My Island* followed, building on the rapport developed, with the women continuing to share and to reassure, support and empathise with each other in relation to various aspects of their lives. Reflection continued in Session 4 with important disclosures around childhood and the women’s past made by all the women. For example, one participant, Helen, talked about her father’s drug and alcohol problems and the stress that had caused her during her childhood, and how she did not want her children growing up seeing what she had seen. Katherine reflected on her own parents and their problems, and how she had never felt loved by them, again emphasising that she did not want to repeat this pattern and wanted her children to be showered by physical affection from her so they would be in no doubt that love was there. The four participants were responsive to each other as they all shared their reflections and empathised with each other in supportive ways [G3obs, grp122, Kathy/Sally facil].

While for some women, sharing the same stage of pregnancy seemed to generate sufficient bonds to reflect comfortably on the past in the context of the group, for others there needed to be more.

Individual women who ‘felt different’ to other participants did not engage with the reflective work:

“I was in such a strange group… In real life I wouldnae speak to this person and I’m no gonna open myself up to this person cause I have got nothing in common with her. She’s on her fourth wean [child]… Then you’ve got super athlete that’s away hiking up the mountains and she’s clearly like really healthy and enjoying pregnancy… one of they people that are just fucking glowing and I’m just no’ in that space.. I’m not going to open myself up unless I know somebody’s dealing with the same kind o’ demons or issues that I’m dealing with… I’ve got to have some commonality with them” [Cecilia, T1part, G2obs. grp116].

Although participants sometimes identified specific characteristics, such as age, as setting them apart, it was more often a sense of them having different lives and experiences which made them reluctant to reflect on their lives in the group.

While several of the women talked about feeling different and distant from other participants, there was one woman interviewed, Isla, who felt that participants in her group [grp106], and to some extent the facilitators too, had distanced themselves from her. Having had several previous children taken away from her and past drug addictions evident in her appearance, she felt she was not being encouraged to share and reflect within the group, either by the facilitators or by the other women. She said she would have been willing, and keen, to do so if she had not picked up on this, but that it constrained her from sharing and reflecting on her past. The accounts of the facilitators and the other group participants, however, suggested that Isla’s own aloofness and reluctance to engage had set her apart. She was ‘the odd one out’–the person who kept leaving to go out for a cigarette–in a group that otherwise formed very strong bonds. One of the facilitators felt that Isla thought her past was “too shocking” to share and reflect on, even though this was a group comprised of extremely vulnerable women whose disclosures included domestic abuse, lack of support and extreme isolation. While Isla did not feel she was different from others in the group, her perception that the others did put her off from participating fully when reflections were shared.

As the example of Isla highlights, the facilitators themselves were seen as a part of the group by the women too. Some of the facilitators drew heavily on their own experience of parenting as a strategy for encouraging participation, contributing to the group as members. This, generally, appeared to put the participants at ease and increased their comfort in reflecting themselves, by the time weeks 3 and 4 were reached. There was minimal criticism of the facilitators by the participants. They tended to like them and the way they delivered MB. Participant engagement did not appear to vary according to who was delivering the programme.

In five of the 28 groups, a problematic participant was identified which limited the participation and/or engagement of other group members in the reflective work. This included: women in two groups who took over and did not give the other women space to reflect themselves [grp101, grp112]; a participant who appeared bored and disinterested whenever others talked about their lives [grp113]; and similarly one who expressed general disinterest and ‘put a dampener’ on the atmosphere, making it difficult for the other women to engage fully [grp114] with *My Island* and *Ghosts from the Past*. There was also a group composed of only two women for Sessions 3 and 4, in which the facilitator felt that one of the women wanted to engage but the other clearly did not, inhibiting the former [Kathy, grp103evfacil].

While low attendance may not have altered how the facilitators implemented the work, some of the women in the groups were there were only one or two participants reported feeling uncomfortable engaging in reflection, or said that the exercises would have worked better if more participants were present:

“we could just concentrate on my problems rather than, like, sharing them but I think it’d be good like if you could speak to different people and see like what they’re experiencing and how they feel” [Gabi, T2part, grp205].

There was also a small number of women who talked about liking the other group members, bonding with them, and feeling part of a coherent group but who did not feel inclined to themselves reflect on their lives. Alex explains that she liked the group because it was up to individual participants how far they wanted to participate in the reflective exercises:

“one of the girls was talking about one thing, it was quite a big thing, whereas other girls have got a sore back, do you know what I mean? So the two problems are like one end of the scale to the other but she still feels like you could speak up with the big problem. It’s pretty open, I feel I could easily speak up [but did not]” [Alex, T1part, grp103].

Future analyses of the THRIVE PE data will explore how these factors influence engagement with MB more generally, but the analyses presented here suggest that group related factors around comfort and bonding are particularly important for engaging the women in the reflective component of MB.

### Mechanisms of change

MB gives participants the opportunity to reflect within the nurturing context of the group in order that maternal stress might be reduced. This is theorised to lead to greater mother-infant attachment. It is also theorised to improve maternal representation of early attachment relationships as a result of this reflection, which will also support greater attachment through improved anticipated outcome of parenting skills and more positive intentions as a mother (see Fig 1). Potential mechanisms include: the sharing of stories, creation of social bonds, the reduction of isolation and developing a greater empathy with one’s bump, all in the context of a nurturing environment.

There was evidence of several mechanisms which might reduce stress and/or support greater attachment: feeling supported/empowered/relief through unburdening and realising others share concerns and/or feeling more in control due to absorbing practical advice around self-care; and increasing empathy with one’s bump. Unintended effects and potentially harmful mechanisms have also been identified, primarily around the reflection working to make some women feel different and more isolated, which may increase maternal stress. The group context, particularly its coherence, appears to be key in understanding whether these mechanisms appear to be inducing change in individual women.

#### Group support

The strongest suggestion of a mechanism of change was through the connections the women formed with other participants, and the feelings of support this engendered. It has already been mentioned that such bonding facilitated engagement with the reflective work, but sharing one’s past also worked to strengthen bonds between the women further, making them feel less isolated. Jenny, for example, was a member of the third group observed [G3obs, grp122]. She was younger than other participants and appeared shy. As part of *My Island* she contributed a jumbled account of her relationship with her father, and detailed a particular childhood incident that continued to affect her. The other women initially seemed to be at a loss as to how to respond, but one empathised and others followed. The researcher recorded:

“Jenny seemed more engaged with all group members at the end and was smiling and making eye contact with people when she was leaving” [G3obs, grp122].

Such bonding and feeling a part of something positive was facilitated not only by reflecting oneself, and receiving a response from the group, but by responding to others and generally “learning more about the girls” [evpart]. Feeling close with others in the group through this sharing was very much valued.

Empowerment was an element of this attachment to the group. Being given the opportunity, having the confidence, and being able to speak freely was powerful in further engendering a sense of involvement. The context of fun and enjoyment in participating in sessions together, even when the subject matter could be serious, appeared to be part of this process of empowerment. Typical comments on *My Island* and *Ghosts from the Past* were:

“As always I feel listened to and that I was given space to talk openly” [evpart]].

“I was given the chance to speak” [evpart].

Relief induced by sharing reflections and, often, realising that one was not alone, was also something talked about by the women:

“[*Ghosts from the Past*] was a really good session when it opened up dialogue. There was only three of us going into things about our past…. I think in pregnancy you start to look at your own childhood…What is good is that you realise that, not necessarily the same, but everyone has fears. Cos you think ‘everyone else is coping so well. Everyone else is doing so well.…’. And that once you’ve sat down and started talking about things then you realise everyone’s just as nervous” [Rita, grp113T2part].

“she [other group member] shared so I should share as well… you can take something from your inside and out it, because when things come outside I think you will feel relaxed… For the lonely person especially it’s really hard to believe and trust all these things. So Mellow Bumps, I think they are doing a great job, they help me…before I was thinking I was the only person having these things. Yes, I met with these ladies and I felt I’m not alone in the world having these problems” [Kala, grp106T1part].

“It was actually quite a relief [to hear other people had hard times]. To think ‘oh, I’m not the only one’. There’s people out there worse than me” [Jane, grp106T1part.

This, in turn, encouraged participants’ own reflection and disclosure, and deeper bonds with fellow participants. They felt they would be listened to and not judged by the other group members.

#### Self-care advice

The facilitator and group response to the reflections of individual women, in terms of teaching them/giving them concrete information about coping strategies and self-care, was another factor highlighted by many of the women as valuable. Learning how to cope with negative thoughts or stress, about the importance of looking after oneself in order to be able to look after the baby, and specific advice tailored to the problems and issues disclosed were all mentioned. In the first group observed [G1obs, grp105], for example, Barbara talked about how she had decided to cease contact with her mother as she had brought too many troubles to her life, largely caused by her long-standing problems with alcohol. Barbara talked about how her feelings of guilt were causing her stress and taking her away from focusing on her own life and parenting responsibilities. Janice and Sally, the facilitators, addressed each issue raised by Barbara in detail and offered practical advice and rationale for focusing on herself.

#### Empathy with bump

In terms of increased empathy with one’s bump, there was less evidence that this was happening. It was mentioned by only a small number of women on the participant evaluation form. Those who did mention it said they appreciated having a greater understanding of how they were parented was shaping how they would parent themselves, and the effect this would have on their child.

#### Unintended effects

There is some suggestion of unintended, and negative, effects of the reflective components of MB for a small number of participants. A small number of women talk about experiencing negative emotions because they felt different or other-ed [37] as a result of disclosures made and/or about being upset by the reflections of others. Again, the key contextual variable was the coherence of the group, with these women being in groups which had not gelled.

For some of the most vulnerable women, feeling that they are different from others in the group could potentially counter many of the beneficial feelings outlined above around feeling a part of something and empowered. For example, Sally outlined how one of the women in a group she facilitated seemed to feel it was important to distance herself from a fellow participant who had disclosed her background of extreme abuse, poverty and deprivation. Although we have no data on how this participant felt, Sally was concerned she would have been hurt or offended by this response:

“you’re there to manage stress during pregnancy, i.e. reducing their cortisol levels. You want to give them the best pregnancy they can have. And really, pulling everything out is not what you want to do” [Sally, T2facil].

Sally did, however, emphasise that this was the only such example of negative effects of the reflective work in the nine groups that she had facilitated. Other examples included reports of groups reacting to the disclosures of others in ‘shocked silence’, apparently at a loss as to how to react. These silences were, however, short-lived and reactions to even the deepest disclosures tended to be non-judgemental, supportive and empathising.

Similarly, Sarah [G1obs, grp105] describes engaging in reflection in the group and feeling she had spoiled the atmosphere in the group as what she talked about was quite shocking. She felt that by making this particular disclosure, she had distanced herself from the rest of the group. Mary [G3obs, grp122] had relatively deep and enduring vulnerabilities in comparison to other participants. Others in the group were supportive, but it was clear by the end of *Ghosts from the Past* that her life was very different to those of the others, prompting questions of how Mary felt about this very obvious discrepancy between her life and the lives of the other participants.

Among those in each group who appeared to have had less troubled pasts there were also a small number of examples of how they found it stressful to hear the reflections of others in the group who had deeper and more enduring vulnerabilities. A participant in one of Sally’s groups, for example:

“had made a big disclosure about something that had happened in her childhood.. [another participant] was not ready for this at all and became very tearful” [Sally, T2facil].

Both participants and facilitators acknowledged that unintended effects might occur as a result of disclosures made within the group, though no other concrete examples were relayed.

There is some evidence to suggest that *some* of the most vulnerable women were less likely to feel a part of a coherent group. This seemed particularly so for the women who had a background of drug addiction and whose appearance belied this, making acceptance into the group difficult. In other cases, the changing dynamics of the group from week to week due to patchy attendance exacerbated the sorts of unintended effects described.

### Outcomes

#### Stress reduction

Although the women rarely talked explicitly about the reflective work reducing their stress levels, it was implicit in much of what was said about its utility (and as highlighted above there was some evidence that it may increase stress levels amongst a small number of women).

Based on these analyses, it seems likely that some of the women who participated in Sessions 3 and 4 of MB, and who were willing and felt able to engage in reflection, may have felt nurtured and relaxed as a result of this. This may have led to some short term reduction in stress. Some of the women who were present in these sessions but who did not feel able to engage in reflection themselves may also have benefited in these ways, by listening to the reflections of others and the nurturing responses. There is little evidence from the PE data, however, of this having more fundamental and longer terms effects on the women such that stress is decreased in more enduring ways. Neither is there evidence to support participants, on the basis of the reflective work alone, improving maternal representation of early attachment relationships to the extent that they improve their anticipated outcome of parenting skills and develop more positive intentions as mothers (though together with other components of MB, this might occur for some participants).

**Fig 2:**
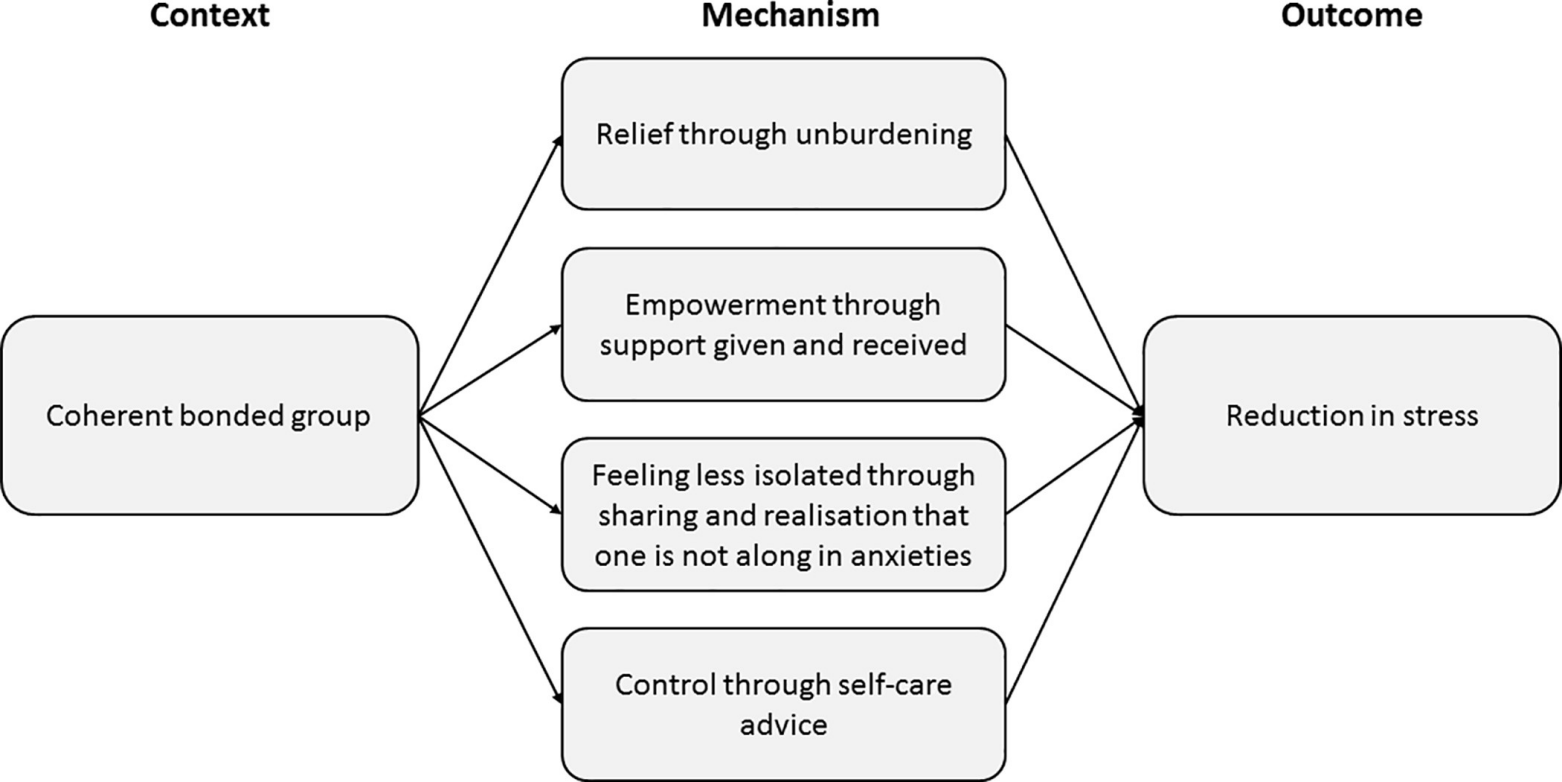
Context, mechanism, outcome configuration.

The group context of MB, for those women who found themselves in a coherent group in which members were able to form strong bonds, appears to be the most important contextual variable underpinning change: how the women feel about themselves as women and parents is more likely to change, for the better, when they find themselves in such coherent supportive groups for the reflective work. Fig 2 outlines the context, mechanism, outcome configuration for the MB reflective work.

## Discussion

This paper has focused on the reflective component of MB exploring how it was implemented and how participants engaged with it in order to identify contextual factors important in understanding mechanisms of change and outcomes. It has found that the reflective exercises were, generally, implemented with fidelity, and that participants tended to engage well with them, although this was dependent on the coherence of the group. Patchy attendance compromised the coherence of some groups, with the development of rapport hindered by varying group composition from week to week. This was seen as a particular problem for the reflective component of MB, where a conducive group atmosphere is crucial for participant comfort. Where there was a coherent group, relief through unburdening, empowerment through support given and received, feeling less isolated through sharing and realisation that one is not alone in anxieties, and control through self-care advice all appeared to be powerful mechanisms of change, leading to stress reduction. Those women with stigmatising, and obvious, vulnerabilities—such as looking like a drug addict–were not always accepted by the group even if the other women established strong bonds. Potentially, stress may be increased for these marginalised women if this makes them feel even more different, and distanced from, their peers.

The analyses highlight why tightly theorised parenting interventions, made up of particular components, might not always be effective in the ways intended when delivered in the real world. It also highlights why some elements of these interventions might work better with some participants rather than others. This might be the case even when they are delivered faithfully. For the reflective component of MB it was the homogeneity of the group–rather than the facilitators’ or participants’ characteristics—that was most important in understanding why some participants did not engage as intended with the reflective exercises.

The importance of feeling part of a supportive group has been highlighted by these analyses. It appears to have empowered participants through their sharing and unburdening, in similar ways reported by other, smaller scale, evaluations of the whole MB programme which have highlighted the importance of social support through group membership [14, 35, 38, 39]. As Birtwell et al found, the MB sessions were a helpful supportive space for sharing emotional experiences [39]. The authors refer to the strength of reflective work, in particular, in enabling the women to come to terms with emotions from the past and manage the anxiety they generate. Birtwell et al identify this as being particularly pertinent during this ante-natal time when the women regard pregnancy as an opportunity to make positive change and repair the past [39]. The wealth of data available for analysis here has enabled a more nuanced account of these sorts of processes identified by these earlier small-scale studies.

There is, however, some evidence in the THRIVE PE data that for some of the most vulnerable participants the process of reflecting may have made them feel even more different from their fellow participants [24]. Westhorp has outlined how and why some early intervention programmes work for most disadvantaged families but not for the most disadvantaged and, indeed, may actually generate negative outcomes for the children in the most disadvantaged families. Westhorp uses context, mechanism and outcome configurations to elucidate this for the interventions she focuses on [24]. The analyses reported here also point to the utility of interrogating how different contexts shape different mechanisms leading to different outcomes, which can help highlight why components of interventions work in different ways for different participants. Further work is needed to explore how the most vulnerable, amongst an already vulnerable group of participants, might benefit as much from group-based reflective work as their peers. For those least vulnerable in this study, there was also a small number whose stress increased because they were exposed to the issues of the most vulnerable. Overall, however, there was much more evidence of beneficial effects of the reflective work than of such harmful effects. Nevertheless, we concur with Bonell et al in urging that such potentially harmful effects of interventions should not be ignored or minimised [40] but rather be better understood through work such as this. Future implementation of MB, and other programmes with a reflective component targeted at vulnerable parents, should incorporate greater awareness of group composition and the backgrounds of individual participants, if possible, and be even more acutely aware of the effect that different levels of disclosure may have on individuals. In order to minimise potential harmful effects, allocation of participants to groups should strive to maximise homogeneity, not only in terms of forms of vulnerability but also levels of vulnerability.

The challenge highlighted by the results presented here is in being able to form and maintain a coherent group. The THRIVE women had many other things happening in their lives. These concerns prevented the women from attending MB on a consistent basis. Skipping sessions occurred despite significant resources being devoted to best practice in maintaining contact with the women throughout the intervention by the facilitators and the THRIVE administrative team; taxis being provided from their home to the venue and back; and snacks being provided during the groups [41] This patchy attendance, combined with some attrition later in the intervention, is consistent with other case studies of the delivery of parenting interventions to vulnerable families [41]. As the reflective component of MB relies on a strongly bonded group as the optimal context for delivery, such a pattern is particularly detrimental.

Intermittent participation was partly attributable to the research context in which MB was implemented. The design of the trial required allocating women to groups that were often not local, involving fairly long journeys and little sense of it being a community-based programme. Furthermore, recruitment challenges meant that some groups started with low numbers. Making access as easy as possible is important. For the THRIVE groups even though transport was organised and paid for, greater proximity might have helped facilitate attendance for those feeling unwell, tired, or balancing childcare demands. What is convenient scheduling for one woman might not be for another [42], and there is a limit to how many groups can be run concurrently for the women at the designated stage of pregnancy: a choice of daytime or evening group would certainly not have been feasible for the THRIVE groups. Unlike the case study described by Schelbe et al, participants were not compensated financially for attending each session [41]. Neither was childcare provided for the THRIVE groups. Such further incentives and potential facilitators of attendance could, perhaps, be considered for future delivery of MB and other interventions with a reflective component, but their cost effectiveness would need to be assessed Schelbe et al suggest addressing ‘inevitable absences’ by reviewing curriculum content at the start of each session [41], but this is unlikely to work for reviewing reflective exercises which rely on the strong rapport compromised by such absences. Neither is one of Schelbe et al’s suggested solutions of replacing weekly sessions with an all-day seminar [41] suited to delivering reflective work where connections are, ideally, developed and strengthened over the weeks. More research is needed to explore how very vulnerable parents can be supported in attending parenting interventions from start to finish.

A limitation of this study is that it does not report outcome data. The trial’s outcomes will be reported at a later date and will clarify whether participants who received MB or ETPB show significantly lower anxiety depression or outwardly directed irritability compared to those receiving CAU alone when their babies are around 6 months old, and whether participants who received MB (plus CAU) show more sensitive interactions with their babies compared to those receiving CAU alone when their babies are around 6 months old [43]. It will be interesting to compare outcomes across women who have and have not received the reflective component. The process data only allow us to explore how the participants and facilitators themselves believe that MB works. Whereas the process data is strong in being able to describe and explain implementation of, and engagement with, the reflective exercises, and show the most likely mechanisms by which they work, we can only hypothesise about their effects. This analysis will feed into better understanding the outcome data, once they become available.

Policy makers should be aware of barriers such as issues around consistent attendance that may compromise the effectiveness of parenting interventions. Retaining those who attend initial sessions should be a priority, with more research needed to understand why some women consistently attend group based parenting programmes such as MB, and others do not. Encouraging continued attendance through ease of access to groups and, perhaps also, incentives is likely to be important. Strategies should be designed which focus on alleviating attendance barriers experienced by those who are likely to drop out. For parenting interventions where part of the theory of change is centred on its delivery in a group context, erosion of that group is likely to compromise at least some components of the intervention. Facilitators should understand its importance as a key contextual variable which will shape outcomes.

## Supporting information

S1 File Mothers interview schedule 1. (PDF)

S2 File Mothers interview schedule 2. (PDF)

S3 File Practitioner interview schedule time 1. (PDF)

S4 File Practitioner interview schedule time 2. (PDF)

S5 File Instructions to researchers observing ETPB and MB group sessions. (PDF)

S6 File Session evaluation form. (PDF)

S7 File Antenatal session evaluation booklet. (PDF)
