# Supplementary material for: The reflective component of the Mellow Bumps parenting intervention: Implementation, engagement and mechanisms of change
Source: PLoS One. 2019 Apr 16;14(4):e0215461. doi: 10.1371/journal.pone.0215461 (PMC6467403; doi:10.1371/journal.pone.0215461)
Supplement: S1 File — (PDF) [file pone.0215461.s001.pdf]

Trial of **H**ealthy **R**elationship **I**nitiatives for the **V**ery **E**arly-years

MOTHERS INTERVIEW SCHEDULE 1

PARTICIPANT INTERVIEW SCHEDULE: TIME 1 OF 2

- To take place towards end of intervention (before baby)
- At 35/36 weeks of pregnancy

SECTION 1: WARM UP

- Tell me about your life:
  - Who you live with
  - How you like to spend your time
  - A little bit about this pregnancy and how it came to happen
  - Relationship with partner
  - Relationship with father of the baby if not current partner
  - Any other children
  - Your wider family.
  - Friends
  - Where live and what kind of home (if not in home)
- How did you come to be taking part in this trial
  - Describe how approached and by who
  - See whether mention vulnerabilities and if did explore a little at this point
- Support during pregnancy
  - SNiPs or equivalent
    - What level of support did you receive (may need prompts regarding contact with health services)
    - Helpfulness of support
    - Benefits of support
  - Contact with other services?
    - How long
    - How that came about
    - How useful was the service
    - Any general comments
- If has not already come up, explore vulnerabilities:
  - Nature of additional need
  - Bit of a history
    - Any planned postnatal support (pre-birth conference)

SECTION 2: RECRUITMENT TO TRIAL

- Re-cap on what said above on this
- Probe:
  - Your understanding of why you were selected to take part
  - Process of first being approached, given any info, told about which arm you were in, what next etc?
  - Their feelings at each stage
    - Enthusiasm
    - Excitement
    - Reluctance
    - Stigma
    - Anxiety
    - Bewilderment
    - Etc

- Did you tell/talk to anyone else
    - What they said
    - Reaction of other
    - If did not talk to anyone why? Because of anticipated reaction?
- How did partner/father of baby/friends/.family/other service contacts feel about involvement or how do you think they feel?
- Explore any barriers re taking part?
- What were your expectations, if any, about the intervention?

SECTION 3: THE INTERVENTION SO FAR

- What do you think about the intervention so far?
- Have you managed to attend all sessions
    - If not, why not?
- Go through each sessions asking what think about it
- Then, probe for
    - Has intervention so far met expectations?
    - Have you enjoyed it? In what ways? Why not?
    - Has it been useful/helpful: in what ways? Why not?
    - Have you felt able to participate fully? If not, why not? Individual sessions?
- Probe on individual sessions, with particular focus on key sessions
    - Explore understanding of what sessions were meant to do/achieve
    - Explore their perception of whether this was achieved
- Anything that, so far, you wish the intervention had done but has not?
- The practitioner: talk about her (him)
    - Whether you like her and why/why not
    - How good at delivering programme
    - How warm/other characteristics
    - Any relationship outside group
- The group: talk about it, describe others, whether work well together
    - Whether like people within it; who, why/why not
    - Has the nature of the group added to or taken away from content?
    - Any relationships outside of group
- The site
    - How convenient has that been to get to
    - How comfortable is room once there
    - Added to or taken away from content of programme
- Travel Provision
    - What has that added to experience, if anything?

SECTION 4: CHILDHOOD

- Explore their experiences of being parented/own childhood
  - What kind of childhood did you have?
    - Family set up
    - How happy
    - Traumatic instances, if any
      - Were there any periods of time when you lived with somebody other than your mother or father?
      - If yes, explore reasons for this, including whether participant was ever in the care system; reason for care entry; thoughts or feelings about being in the care system; memories of who you lived with; have you remained in contact with them and why/why not?
      - If no, explore same as above, but focussing on who that person was and why they lived there… i.e. was it an informal care arrangement.
    - Relationship with parents
      - Specifically if in care system or lived with other adult during childhood… what was contact with parents (if alive) like during that period and how did that affect long-term relationship with parents?
      - Were there differences in parenting styles, and if so, in what way? How did that make them feel etc.?
    - Other close people to them
  - Idea of people sometimes revisiting their childhood when you find out that you are pregnant – did you find yourself reflecting on this when found out you were pregnant this time, or previous pregnancies
  - Would you like to be a mother like her mother was? In what ways? In what ways not? (and father too)
  - What is a 'good mother'?
  - Was your mother a 'good mother' – in what ways yes, in what ways no
  - **If** in care system or lived with other adult during childhood have your views on motherhood/parenting been based on how you were parented by other people? What was good/bad? What would you do same/differently?
  - What are your hopes for motherhood?
  - To what extent has the intervention got you to think more about these kinds of things?
  - In what ways, if any, has it been helpful?
  - If any worries expressed around negative bits of childhood and own parenting, explore this more – what can help this not happening again? Explore nature of anxieties? Explore extent to which intervention has helped or not?
