# Supplementary material for: The reflective component of the Mellow Bumps parenting intervention: Implementation, engagement and mechanisms of change
Source: PLoS One. 2019 Apr 16;14(4):e0215461. doi: 10.1371/journal.pone.0215461 (PMC6467403; doi:10.1371/journal.pone.0215461)
Supplement: S2 File — (PDF) [file pone.0215461.s002.pdf]

Trial of **H**ealthy **R**elationship **I**nitiatives for the **V**ery **E**arly-years

MOTHERS INTERVIEW SCHEDULE 2

PARTICIPANT INTERVIEW SCHEDULE TIME 2 OF 2

- Further informed by data collection in meantime, as whole and from individual mums (preliminary analysis will have taken place, including of the time 1 in-depth interviews with the same mothers).
- Will take place when the child(ren) is 6 months old.
-

SECTION 1: UPDATE CIRCUMSTANCES

- o How has life been since last interview (and since baby born)
- o Talk about changes/major life events since last interview
- o Tell me all about the baby and how you are getting on
- o Generally, what is life like?

SECTION 2: RECAP (VERY BRIEF) ON WHAT THEY SAID ABOUT THE INTERVENTION AT TIME 1 AND UPDATE

- o Explore impressions of the last part of the intervention, delivered since Time 1 interview; how do you rate its usefulness/enjoyability
- o Generally, what do you remember about:
  - the intervention
  - Practitioner?
  - Group?
- o Legacy of intervention
  - Which bits have been useful – concrete examples
- o Anything that you now wish had been covered
- o Did the intervention improve/change your:
  - Knowledge/understanding of infant needs (how)
  - Attitudes regarding being a parent/child rearing (how)
  - Behaviour regarding parenting/skills to engage with baby and respond to needs
  - Behaviour regarding partner/father of baby (and his behaviour towards you)
  - Feelings: self-awareness of how you feel re baby
- o Talk about any changes in the following *as a result of the intervention*
  - Self-esteem
  - Self-confidence generally and as a parent
  - Anxiety generally and as a parent
  - Self-accepting generally and as a parent
  - Feelings of guilt generally and as a parent

SECTION 3: Since birth of baby, extent to which thought about own childhood and how parented

- o Reflections on this (informed by T1 interview)
