# Supplementary material for: The reflective component of the Mellow Bumps parenting intervention: Implementation, engagement and mechanisms of change
Source: PLoS One. 2019 Apr 16;14(4):e0215461. doi: 10.1371/journal.pone.0215461 (PMC6467403; doi:10.1371/journal.pone.0215461)
Supplement: S3 File — (PDF) [file pone.0215461.s003.pdf]

Trial of **H**ealthy **R**elationship **I**nitiatives for the **V**ery **E**arly-years

PRACTITIONER INTERVIEW SCHEDULE TIME 1

SECTION 1: WARM UP

Tell me about your professional background

- How did you get to where you are today?
- Previous roles and how relate to current role

SECTION 2: PREVIOUS EXPERIENCE WORKING WITH VULNERABLE FAMILIES

What experience do you have working with vulnerable families?

What would be your ‘normal’ care plan for vulnerable woman?

- What organisations would be involved in their care?
    - Statutory
    - Third sector?

SECTION 3: UNDERSTANDINGS OF THE PROJECT

Before you heard about this project, were you aware of either of the antenatal programmes

- Previous experiences
- Perceived benefits or drawbacks?
    - Any concerns?

Did you feel happy to refer to the project?

- Did you feel you had sufficient information?
- Types of women you referred
- Were there many women you approached and they declined to take part?
    - Did they give any reasons?
- Was there anyone you chose not to refer?
    - Why?
- Were there any barriers to referral?
    - Appropriateness of time frame
    - User-friendliness of referral process

SECTION 4: ATTITUDES ABOUT RESEARCH

Have you had any previous experience of being involved with a research project?

- Perceived benefits or drawbacks?
- Any concerns?
