# Supplementary material for: The reflective component of the Mellow Bumps parenting intervention: Implementation, engagement and mechanisms of change
Source: PLoS One. 2019 Apr 16;14(4):e0215461. doi: 10.1371/journal.pone.0215461 (PMC6467403; doi:10.1371/journal.pone.0215461)
Supplement: S4 File — (PDF) [file pone.0215461.s004.pdf]

Trial of **H**ealthy **R**elationship **I**nitiatives for the **V**ery **E**arly-years

PRACTITIONER INTERVIEW SCHEDULE TIME 2

PRACTITIONER INTERVIEW SCHEDULE: TIME 2 OF 2

To take place:

- after completion of T3 questionnaire
- 12 months after training has been delivered

SECTION 1: UPDATE CIRCUMSTANCES

- Talk about employment changes since last interview

SECTION 2: RUNNING THE INTERVENTIONS

- Have you delivered an intervention group? If not, why not?
- Are you still delivering intervention groups? If not, why not?
- Roughly how many sessions of X intervention have you delivered?
  - Do you have other roles? What are they? How balancing commitments?
- Explore understanding of what the sessions consist of
  - Probe on individual sessions, with particular focus on key sessions
    - Explore understanding of what sessions were meant to do/achieve
    - Explore perception of whether this was achieved
  - Which sessions do mothers/fathers respond best to? Why?
  - Which sessions do mothers/fathers not respond to? Why?
  - Where there any sessions that were challenging to deliver? Why?
- Did you face any challenges when running the group sessions?
  - Location and suitability of venue
  - Distance to venue and transport
  - Timings of sessions
  - Group dynamics: Where women able to participate fully? If not, why not?
  - Relationship with other practitioner
    - Did you get along? Challenges of working together; preferred lone working?
    - How did you share delivery of sessions?
- ETPB only: what about individual sessions?
  - Did all women engage equally?
  - Barriers to mother's engagement
    - Support, timing, other children etc.
- Have you enjoyed delivering the sessions? If not, why not?
- Would you change anything about the interventions? If so, what and why?
  - Related: Were there parts of the course you didn't like delivering? If not, why not?
  - Related: Anything that, so far, you wish the intervention had done but has not?
  - Explore how closely the course content was adhered to?
    - Did you change any parts of the course, either by omitting/adding material?

- If made changes, what were those changes and why were these made?
  - Not enough time
  - Personal attitudes/beliefs
  - Based on women's responses, comprehension etc.
  - Other reasons
- If not, do you think there might be a time where you would deliver the packages differently? Why? To whom? In what circumstances?

- Were there any barriers to women engaging in the sessions? If so, what?
  - Trust, suspicion, didn't feel service was needed, no support from partner etc.
  - Differences between those who attended all the sessions vs. some of the sessions?
- Who do they think group/individual sessions worked best for?
  - Different vulnerabilities
  - Amount of support women received
- Did any of the mother's receiving intervention become "dependent" on you as practitioner?
  - What characteristics associated with this?
  - How did you promote autonomy, self-reliance etc.

SECTION 3: TRAINING

- Explore impressions of training & trainer
  - Whether you like him/her and why/why not
  - How good at delivering training
  - How warm/other characteristics
  - Any relationship with trainer outside group
- What did the training involve?
- Recap expectations of training raised in previous interview
  - Did training meet expectations?
    - Did you enjoy it? In what ways? Why not?
    - Were you able to fully participate in training sessions. If not, why not?
    - Has it been useful/helpful: in what ways? Why not?
    - Did it affect confidence to deliver group sessions? If not, why not?
    - What about Individual sessions? If not, why not?
- Summarise challenges of delivering groups.
  - Did training prepare them for those?
  - Help them overcome them
- Could training have been improved?
  - How?

SECTION 4: SUPERVISION

- Explore impressions of supervisor(s)
  - Whether you like him/her and why/why not?
  - How good were they at delivering supervision
    - Do they help you address problems, finds solutions etc.
    - Are they supportive of practitioners? If so, how? If not, in what way?
    - How do they view the mothers/fathers and their circumstances
      - Empathy
      - Respect
      - Support needed
      - Anything else
    - Does their view of mothers/fathers affect the quality of their supervision? If so, in what way?
- What do you think about the supervision you have received?
  - Helpful? If so, in what way? If not, why not and how could it be improved?
    - Supportive?
    - Useful?
    - Pastoral/emotional support?
    - Problem solving?
    - Other?
  - How warm/other characteristics
  - Any relationship with supervisor outside group
- Could supervision be improved?
  - How?
