# Supplementary material for: The reflective component of the Mellow Bumps parenting intervention: Implementation, engagement and mechanisms of change
Source: PLoS One. 2019 Apr 16;14(4):e0215461. doi: 10.1371/journal.pone.0215461 (PMC6467403; doi:10.1371/journal.pone.0215461)
Supplement: S5 File — (PDF) [file pone.0215461.s005.pdf]

Trial of **H**ealthy **R**elationship **I**nitiatives
for the **V**ery **E**arly-years

INSTRUCTIONS TO RESEARCHERS OBSERVING ETPB AND MB GROUP SESSIONS

Please write detailed notes regarding how the women interact with each other, how the practitioner interacts with the women, and vice versa, level and nature of participation, practitioner style, comfort of room and facilities.

The following should be noted systematically:

- What session is being delivered: content, including how closely complies with MB/ETBB packs
- Who is delivering it
- Style of practitioner
- Relationship practitioner-women
- How many women in attendance
- Do women interact with each other/part of group/not
- Do all women participate fully
- Attitudes of women (eg enjoyment, interest, engagement, disruption etc)
- Evidence of learning
- Evidence of attitude change
- Evidence of skills development
