# Supplementary material for: The reflective component of the Mellow Bumps parenting intervention: Implementation, engagement and mechanisms of change
Source: PLoS One. 2019 Apr 16;14(4):e0215461. doi: 10.1371/journal.pone.0215461 (PMC6467403; doi:10.1371/journal.pone.0215461)
Supplement: S6 File — (PDF) [file pone.0215461.s006.pdf]

Trial of **H**ealthy **R**elationship **I**nitiatives for the **V**ery **E**arly-years

SESSION EVALUATION FORM

Let us know what you thought about today's session

Session No. ____________________

Date

d	d	m	m	y	y	y	y

How much do you agree with the following statements?

Please tick one box per line

	Strongly disagree	Disagree	Neither agree or disagree	Agree	Strongly agree
The session was well prepared	❐ 0	❐ 1	❐ 2	❐ 3	❐ 4
The person explained things well	❐ 0	❐ 1	❐ 2	❐ 3	❐ 4
The room was comfortable	❐ 0	❐ 1	❐ 2	❐ 3	❐ 4
I felt like I could speak when I wanted to	❐ 0	❐ 1	❐ 2	❐ 3	❐ 4
The session was too long	❐ 0	❐ 1	❐ 2	❐ 3	❐ 4
I was bored	❐ 0	❐ 1	❐ 2	❐ 3	❐ 4
I had fun	❐ 0	❐ 1	❐ 2	❐ 3	❐ 4
Time went fast	❐ 0	❐ 1	❐ 2	❐ 3	❐ 4
The materials were useful	❐ 0	❐ 1	❐ 2	❐ 3	❐ 4
I learned a lot	❐ 0	❐ 1	❐ 2	❐ 3	❐ 4
I felt listened to	❐ 0	❐ 1	❐ 2	❐ 3	❐ 4
I felt left out	❐ 0	❐ 1	❐ 2	❐ 3	❐ 4

What did you **like** about today's session?

What did you **not like** about today's session?

What would have made it better?

Is there anything else you would like to say?
