# Supplementary material for: The reflective component of the Mellow Bumps parenting intervention: Implementation, engagement and mechanisms of change
Source: PLoS One. 2019 Apr 16;14(4):e0215461. doi: 10.1371/journal.pone.0215461 (PMC6467403; doi:10.1371/journal.pone.0215461)
Supplement: S7 File — (PDF) [file pone.0215461.s007.pdf]

Trial of **H**ealthy **R**elationship **I**nitiatives for the **V**ery **E**arly-years

Antenatal Session Evaluation Booklet

Which parenting programme did you deliver?

Enhanced Triple P for Baby ☐

Mellow Bumps ☐

PLACE BARCODE HERE
CHECK MATERIAL CODE CORRECT

Practitioner name: ....

It would be helpful if you could use this booklet to write notes about the group sessions. We would like to know a little about how the sessions went and how you felt about it

Please use the attendance form to record each woman's attendance in the group sessions

Enhanced Triple P for Baby Practitioners should complete Session 1-4 only

Guidance:

Length: of session

Focus: Key topics covered

Evaluation: Please think about and comment on:

- Your overall impression of the session?
- What went well? What didn't go so well? Any reason?
- Do you feel that participants are engaged with the programme equally or are some people more engaged than others? Any reason?
- How did the participants relate to each other?
- Is this group easy or challenging to work with? Any reason?

Rating: Please rate who you felt the session went **-** 5 being the highest and 1 the lowest score. For example if you felt that the session went very well – the participants seemed engaged with the programme and you were able to talk about all the key topics as planned – the score would be 5. However, if the session went badly – the participants seemed disinterested in the programme and you were not able to talk about any of the key topics as planned – you might score 1.

Comments: any information that you feel is relevant

There is space at the end of this booklet for you to add extra comments.

Please use this form to keep a record of the women who attend each week.

Please enter a tick under the week if the woman has attended the session.

Enhanced Triple P for Baby Practitioners should complete Week 1-4 and record partner attendance by adding a '+1' to the box beside the tick.

Name	Taxi required?	Week 1	Week 2	Week 3	Week 4	Week 5	Week 6	Week 7

**Session No**	**1**				
**Date**					
**Length of session** *(minutes)*					
**Focus**<br>*(Key topics covered)*					
**Was there anything you didn't cover?**					
**Which elements did the participants engage with best and worst? Any reason?**					
**Evaluation of session**<br>*(What was your overall impression of the session? What went well? What didn't go so well? Any reason?*<br>*Do you feel that participants are engaged with the programme equally or are some people more engaged than others? Any reason?*<br>*How did the participants relate to each other?*<br>*Is this group easy or challenging to work with? Any reason?)*					
**Overall rating of session**	1	2	3	4	5
**Comments**					

**Session No**	**2**				
**Date**					
**Length of session** *(minutes)*					
**Focus** *(Key topics covered)*					
**Was there anything you didn't cover?**					
**Which elements did the participants engage with best and worst? Any reason?**					
**Evaluation of session** *(What was your overall impression of the session? What went well? What didn't go so well? Any reason? Do you feel that participants are engaged with the programme equally or are some people more engaged than others? Any reason? How did the participants relate to each other? Is this group easy or challenging to work with? Any reason?)*					
**Overall rating of session**	1	2	3	4	5
**Comments**					

**Session No**	**3**				
**Date**					
**Length of session** *(minutes)*					
**Focus** *(Key topics covered)*					
**Was there anything you didn't cover?**					
**Which elements did the participants engage with best and worst? Any reason?**					
**Evaluation of session** *(What was your overall impression of the session? What went well? What didn't go so well? Any reason? Do you feel that participants are engaged with the programme equally or are some people more engaged than others? Any reason? How did the participants relate to each other? Is this group easy or challenging to work with? Any reason?)*					
**Overall rating of session**	1	2	3	4	5
**Comments**					

<table>
<tr><td>Session No</td><td colspan="5">4</td></tr>
<tr><td>Date</td><td colspan="5"></td></tr>
<tr><td>Length of session
(minutes)</td><td colspan="5"></td></tr>
<tr><td>Focus
(Key topics covered)</td><td colspan="5"></td></tr>
<tr><td>Was there anything you didn’t cover?</td><td colspan="5"></td></tr>
<tr><td>Which elements did the participants engage with best and worst? Any reason?</td><td colspan="5"></td></tr>
<tr><td>Evaluation of session
(What was your overall impression of the session? What went well? What didn’t go so well? Any reason?
Do you feel that participants are engaged with the programme equally or are some people more engaged than others? Any reason?
How did the participants relate to each other?
Is this group easy or challenging to work with? Any reason?)</td><td colspan="5"></td></tr>
<tr><td>Overall rating of session</td><td>1</td><td>2</td><td>3</td><td>4</td><td>5</td></tr>
<tr><td colspan="6">Comments</td></tr>
</table>

**Session No**	**5**				
**Date**					
**Length of session** *(minutes)*					
**Focus** *(Key topics covered)*					
**Was there anything you didn't cover?**					
**Which elements did the participants engage with best and worst? Any reason?**					
**Evaluation of session** *(What was your overall impression of the session? What went well? What didn't go so well? Any reason? Do you feel that participants are engaged with the programme equally or are some people more engaged than others? Any reason? How did the participants relate to each other? Is this group easy or challenging to work with? Any reason?)*					
**Overall rating of session**	1	2	3	4	5
**Comments**					

**Session No**	**6**				
**Date**					
**Length of session** *(minutes)*					
**Focus** *(Key topics covered)*					
**Was there anything you didn't cover?**					
**Which elements did the participants engage with best and worst? Any reason?**					
**Evaluation of session** *(What was your overall impression of the session? What went well? What didn't go so well? Any reason? Do you feel that participants are engaged with the programme equally or are some people more engaged than others? Any reason? How did the participants relate to each other? Is this group easy or challenging to work with? Any reason?)*					
**Overall rating of session**	1	2	3	4	5
**Comments**					

**Session No**	**7**				
**Date**					
**Length of session** *(minutes)*					
**Focus** *(Key topics covered)*					
**Was there anything you didn't cover?**					
**Which elements did the participants engage with best and worst? Any reason?**					
**Evaluation of session** *(What was your overall impression of the session? What went well? What didn't go so well? Any reason? Do you feel that participants are engaged with the programme equally or are some people more engaged than others? Any reason? How did the participants relate to each other? Is this group easy or challenging to work with? Any reason?)*					
**Overall rating of session**	1	2	3	4	5
**Comments**					

Additional comments:
